# Supplementary material for: Clinical Significance of Ventricular Premature Contraction Provoked by the Treadmill Test
Source: Medicina (Kaunas). 2022 Apr 18;58(4):556. doi: 10.3390/medicina58040556 (PMC9028355; doi:10.3390/medicina58040556)
Supplement: Supplementary file 1 [file medicina-58-00556-s001.zip › medicina-1647386-supplementary.pdf]

Supplement Table S1. Clinical Correlation in exercise provoked VPC group (n=200, multivariable)

	VPC_Y (n=32)	VPC_E (n=21)	VPC_R (n=20)
Age		1.031 (0.995-1.068)	
FHxCAD		3.407 (0.968-11.995)	
FHxSCD	**5.786 (1.111-30.120)**		**10.875 (2.026-58.363)**

Data are presented as odds ratio (95% confidence interval)

Included variable: age, sex, BMI, FHxCAD, FHxSCD, DM, HTN, maximal HR during exercise, maximal SBP during exercise.

Abbreviations: abbreviations are listed in Table 1.

The values in bold indicate statistical significance ($p<0.05$).

Supplement Table S2. Baseline characteristics of VPC_E (n=21) and VPC_R (n=20)

	VPC_E (n=21)	VPC_R (n=20)	P value (VPC_E vs VPC_R)
**Demographics**			
Age	57.7 ± 16.1	53.8 ± 17.9	0.465
Sex (male)	3 (14.3%)	5 (25.0%)	0.454
BMI	25.2 ± 2.8	24.5 ± 2.7	0.429
**Pre. Medical Hx**			
DM	3 (14.3%)	3 (15.0%)	1.000
HTN	9 (42.9%)	9 (45.0%)	1.000
Dyslipidemia	3 (14.3%)	6 (30.0%)	0.277
CVA	2 (9.5%)	2 (10.0%)	1.000
PAD	2 (9.5%)	2 (10.0%)	1.000
**Exercise Test**			
Maximal HR	154.1 ± 20.7	158.1 ± 24.7	0.580
Maximal SBP	190.8 ± 35.0	185.9 ± 33.5	0.652
Exercise capacity	11.4 ± 2.0	11.2 ± 2.7	0.788
TMT positive	5 (23.8%)	3(15.0%)	0.697

Data are presented as mean ± standard deviation or n (%).

Abbreviations: abbreviations are listed in Table 1.
